# White Matter Organization and Cortical Thickness Differ Among Active Duty Service Members With Chronic Mild, Moderate, and Severe Traumatic Brain Injury

**DOI:** 10.1089/neu.2023.0336

**Published:** 2024-04-04

**Authors:** Sarah I. Gimbel, Lars D. Hungerford, Elizabeth W. Twamley, Mark L. Ettenhofer

**Affiliations:** ^1^ Traumatic Brain Injury Center of Excellence, Silver Spring, Maryland, USA.; ^2^ Naval Medical Center San Diego, San Diego, California, USA.; ^3^ General Dynamics Information Technology, Falls Church, Virginia, USA.; ^4^ University of California, San Diego, San Diego, California, USA.; ^5^ Center of Excellence for Stress and Mental Health, VA San Diego Healthcare System, San Diego, California, USA.

**Keywords:** cortical thickness, diffusion tensor imaging (DTI), fractional anisotropy (FA), magnetic resonance imaging (MRI), pothole analysis, traumatic brain injury (TBI)

## Abstract

This study compared findings from whole–brain diffusion tensor imaging (DTI) and volumetric magnetic resonance imaging (MRI) among 90 Active Duty Service Members with chronic mild traumatic brain injury (TBI; *n* = 52), chronic moderate-to-severe TBI (*n* = 17), and TBI-negative controls (*n* = 21). Data were collected on a Philips Ingenia 3T MRI with DTI in 32 directions. Results demonstrated that history of TBI was associated with differences in white matter microstructure, white matter volume, and cortical thickness in both mild TBI and moderate-to-severe TBI groups relative to controls. However, the presence, pattern, and distribution of these findings varied substantially depending on the injury severity. Spatially-defined forms of DTI fractional anisotropy (FA) analyses identified altered white matter organization within the chronic moderate-to-severe TBI group, but they did not provide clear evidence of abnormalities within the chronic mild TBI group. In contrast, DTI FA “pothole” analyses identified widely distributed areas of decreased FA throughout the white matter in both the chronic mild TBI and chronic moderate-to-severe TBI groups. Additionally, decreased white matter volume was found in several brain regions for the chronic moderate-to-severe TBI group compared with the other groups. Greater number of DTI FA potholes and reduced cortical thickness were also related to greater severity of self-reported symptoms. In sum, this study expands upon a growing body of literature using advanced imaging techniques to identify potential effects of brain injury in military Service Members. These findings may differ from work in other TBI populations due to varying mechanisms and frequency of injury, as well as a potentially higher level of functioning in the current sample related to the ability to maintain continued Active Duty status after injury. In conclusion, this study provides DTI and volumetric MRI findings across the spectrum of TBI severity. These results provide support for the use of DTI and volumetric MRI to identify differences in white matter microstructure and volume related to TBI. In particular, DTI FA pothole analysis may provide greater sensitivity for detecting subtle forms of white matter injury than conventional DTI FA analyses.

**Figure:**
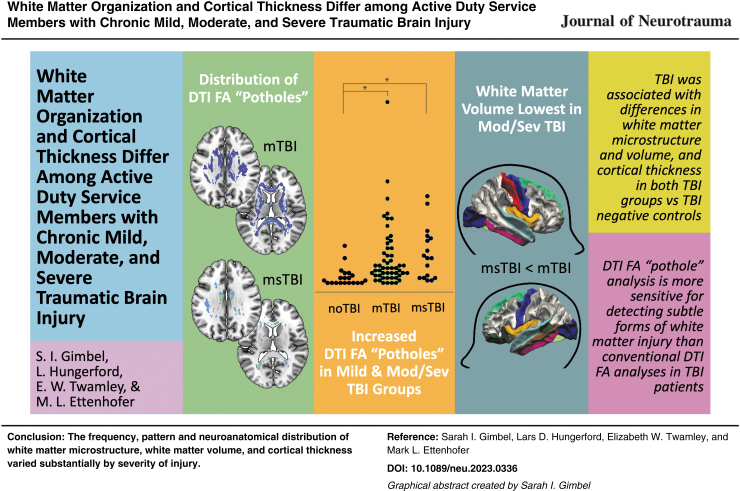


## Introduction

More than 473,000 United States Military Service Members have been diagnosed with traumatic brain injury (TBI) since 2000. The majority of these injuries are classified as “mild” (mTBI), with roughly 12% classified as “moderate” or “severe” (msTBI).^1^ Brain computed tomography and conventional magnetic resonance imaging (MRI) results are often negative after TBI, especially for those with mTBI. However, based on findings from advanced forms of imaging, cortical thinning and diffuse axonal injury (a shearing injury in which the white matter of the brain is damaged) are common following TBI and are thought to underlie many persistent cognitive impairments.^2-4^ Diffusion tensor imaging (DTI), which is often used to characterize white matter microstructure in the form of fractional anisotropy (FA), has grown in popularity as a method for detecting injury-related abnormalities that may not be visible on commonly used clinical scans such as T1 or T2 imaging.^5-7^ However, given the heterogenous spatial distribution of white matter pathology in TBI, voxel-wise and tractographic methods for examining white matter organization in group data may not have sufficient power to truly reflect the effects of TBI on white matter organization.

In the general population, many studies have examined white matter organization across the spectrum of TBI severity, both in the acute (< 3 month) and chronic (> 3 month) phases of injury. Changes in white matter organization have been documented as early as the day of injury^8,9^ and often persist for many years.^10–14^ Midline brain structures such as the corpus callosum are believed to be most susceptible to the shearing and rotational forces of traumatic brain injury.^15-17^ Indeed, a landmark postmortem study reported damaged axons in the corpus callosum and fornix following TBI, providing evidence that these regions are most vulnerable to trauma;^18^ subsequent in vivo studies have supported this finding.^11,19^ Additionally, other major white matter structures including the internal capsule,^11^ superior and inferior longitudinal fasciculi,^19^ and cerebellar fibers^20,21^ have commonly shown abnormalities following TBI.

A recent meta-analysis of white matter organization following TBI confirmed that DTI findings can differ substantially for mild versus moderate/severe TBI populations.^22^ For those who have suffered msTBI, DTI studies reliably reveal decreased FA persisting into the chronic stage of injury, suggesting loss of axonal integrity, specifically in the corpus callosum, internal capsule, and superior longitudinal fasciculus,^11^ as well as other long and short white matter fibers.^13^ Although variability exists in the tracts demonstrating abnormalities following mTBI, there are some consistent findings as well. Many mTBI studies have demonstrated white matter abnormalities in the acute and subacute stages of injury,^23^ but in a subset of individuals, these deficits may persist many years post-injury.^24-26^ Similar to the msTBI group, white matter changes in the mTBI group are most frequently identified in commissural and long coursing fiber tracts, albeit typically to a lesser extent.^11,27^ Anterior corona radiata, forceps major, and corpus callosum have shown increased loss of integrity in the chronic stage of mTBI recovery, predicting the persistence of post-concussion syndromes.^28^ Thus, certain tracts may provide an objective biomarker for tracking the pathological recovery process following mTBI.

There is, however, less anatomical consistency for injured regions in mTBI compared with the msTBI population, possibly related to differences in mechanisms and type of injury. While one study of chronic stage mTBI found no difference in corpus callosum FA between the mTBI and non-brain injured control groups,^29^ a meta-analysis including both acute and chronic studies concluded that the corpus callosum is indeed vulnerable in mTBI, with no significant moderation of this effect based on time since injury.^30^

The lack of consistent findings at a sub-regional level in chronic TBI is likely related to the diffuse and heterogeneous spatial distribution of injury in the brain, especially in the mild group. One approach to address this issue, known as “pothole” analysis, identifies heterogeneous areas of decreased FA using voxel-wise comparison of an injured individual's FA to a reference control population. In a study of veterans with mTBI, Jorge and colleagues found no voxel-wise differences between the mTBI group and a healthy control group in FA, but the mTBI group had a greater number of potholes across the brain than the control group.^31^ However, relatively few studies to date have used this approach for examining white matter abnormalities, despite consensus that standard practices for examining FA in a heterogeneous group of TBI patients may be inadequate.

While significantly less is known about cortical structure changes following TBI, early understanding comes from animal models, which have showed cortical thickening (likely due to edema) immediately following brain injury, followed by thinning over time.^32^ Since most mTBI patients fail to show detectible cortical abnormalities using traditional neuroradiological examinations, more advanced cortical segmentation, with inspection of gray matter thickness, may elucidate connections between cortical findings following TBI and sustained effects of injury.^33^

A study of veterans in the chronic stage of mTBI (>3 months post-injury) showed thinning of cortex in the left dorsal superior frontal and superior temporal gyri, signifying early effects of mTBI in the cerebral cortical architecture.^34^ In a longitudinal study following patients from acute injury to 3 months post-injury, patients showed significant cortical thinning in the middle temporal gyrus compared with orthopedic-injury controls.^35^ An additional study of sports-related mTBI found cortical thinning in the left dorsolateral prefrontal cortex and right inferior parietal cortex compared with uninjured participants.^36^ Although many studies have shown a variety of regional changes in cortical thickness, including evidence for frontotemporal vulnerability following TBI,^37–48^ no consistent findings across studies have emerged. Additionally, a number of recent studies have reported no differences in cortical thickness between brain injured and uninjured groups,^49-51^ limiting the generalizability of differences that have been identified in this population.

These changes in white matter organization and cortical thickness following TBI may be understood in the context of previous findings related to cellular neuropathology. TBI is known to trigger the release of pro-inflammatory cytokines and chemokines, which can activate the brain's immune response in both microglia and astrocytes. This can then lead to the release of additional pro-inflammatory molecules, creating a cycle of inflammation that can cause neuronal damage.^52^ This response can contribute to neuronal and glial cell death as well as the disruption of the blood–brain barrier, exacerbating inflammation and leading to tissue damage.^53^ Damaged fiber tracts and disruption of myelin surrounding neurons can lead to cellular loss, ultimately expressed as volume loss in the brain.^54^ In sum, previous findings related to the cellular neuropathology of TBI underscore the need to examine both white matter organization and gray matter thickness when seeking to understand brain markers of injury.

This study was conducted to evaluate structural white matter and gray matter changes in Active Duty Service Members with a history of chronic/symptomatic TBI (mild, moderate, or severe). These participants were compared with a group of healthy control Service Members with no history of brain injury. A goal of this study is to use the same procedures to examine white and gray matter integrity across the TBI severity spectrum, comparing mild, moderate/severe, and uninjured groups all within an Active Duty Service Member population. Mild and moderate/severe injury groups were evaluated separately to identify differential brain structure changes related to injury severity. Moderate and severe TBI groups did not differ in white matter microstructure or gray matter cortical thickness, and thus were grouped together for analysis. Voxel-wise and region of interest (ROI)–based analyses of FA and cortical thickness were performed in conjunction with the more novel pothole analysis approach to increase sensitivity for identifying potential lasting effects of TBI in an Active Duty population.

## Methods

### Participants

Participants included U.S. Active Duty military volunteers recruited from military treatment facilities in the San Diego area. The TBI groups consisted of adults (>18 years old) with persistent self-reported symptoms related to TBI sustained more than 3 months before study participation; the control group consistent of adults with no history of TBI or other neurological conditions (noTBI). Participants were divided into groups based on VA/DOD guidelines, defining mild TBI as <30 min loss of consciousness, <24 h confusion/disorientation, and/or <24 h post-traumatic amnesia; moderate TBI as 30 min-24 h loss of consciousness, >24 h confusion/disorientation, and/or >24 h post-traumatic amnesia; and severe TBI as >24 h loss of consciousness, GCS 3-8, and/or >7 days post-traumatic amnesia. Ninety participants (*n* = 21 noTBI, *n* = 52 mTBI, *n* = 17 msTBI) met full eligibility requirements and were included in analysis. After determining that there were no significant differences in brain metrics between the two groups, the moderate (*n* = 12) and severe (*n* = 5) TBI groups were combined for the purposes of this study. Participants were excluded from this study if they had a neurological condition, psychiatric disorders, contraindications for MRI, or a history of a medical condition that would be expected to affect cognitive or motor abilities.

### Experimental procedure

After providing written informed consent, participants provided demographic information and medical history (Table 1). History of TBI was obtained using the Ohio State University TBI Identification Method (OSU TBI-ID)^55,56^ and verified using available medical records. Participants completed a brief screening battery of standardized self-reported symptom surveys, including Neurobehavioral Symptom Inventory (NSI),^57^ PCL-5 (Post-Traumatic Stress Disorder Checklist for Diagnostic and Statistical Manual of Mental Disorders, Fifth Edition),^58^ and Headache Impact Test-6 (HIT6).^59-61^ Participants then completed structural and functional MRI. This research was approved by the Institutional Review Board at Naval Medical Center San Diego.

**Table 1.** Demographic Information and Self-Report Scores

	noTBI	mTBI	msTBI	*p* ^a^
*n*	21	52	17	-
Sex	14M, 7F	42M, 10F	15M, 2F	.193
Age (years)	29.62 ± 7.28	35.00 ± 7.81	26.41 ± 7.88	<.001^*^
Number of lifetime TBIs	-	3.92 ± 2.46 (max: 13, min: 1)	1.94 ± 2.19 (max: 10, min: 1)	.004^*^
Time since last TBI (months)	-	48.04 ± 44.19	10.29 ± 18.30	.001^*^
Cause of most recent TBI	-			
Motor vehicle accident		13 (25%)	5 (29%)	
Military training/deployment		17 (33%)	1 (6%)	.034^*^
Fall/accident		13 (25%)	9 (53%)	
Sports		7 (13%)	-	
Assault/abuse		2 (4%)	2 (12%)	
Race/ethnicity				
White	13 (62%)	35 (67%)	8 (47%)	
Hispanic	5 (24%)	6 (11%)	4 (24%)	.619
Asian	0	2 (4%)	1 (6%)	
Black/African American	0	4 (8%)	3 (17%)	
Native Hawaiian/Pacific Islander	1 (5%)	2 (4%)	0	
American Indian/Alaska Native	0	1 (2%)	0	
Other	2 (9%)	2 (4%)	1 (6%)	
U.S. Military Branch of Service				
Army	1 (5%)	3 (6%)	0	
Marine Corps	1 (5%)	2 (4%)	6 (35%)	.020^*^
Navy	19 (90%)	43 (82%)	11 (65%)	
Air Force	0	2 (4%)	0	
Coast Guard	0	2 (2%)	0	
Years of education	14.83 ± 2.79	14.75 ± 2.23	13.00 ± 1.54	.018^*^
NSI-22 Total Score	8.52 ± 12.46	33.10 ± 18.53	18.06 ± 15.98	<.001^*^
PCL-5 Total Score	9.33 ± 15.13	30.36 ± 20.91	21.24 ± 19.74	<.001^*^
HIT-6 Total Score	4.81 ± 4.39	35.64 ± 26.50	6.24 ± 7.41	<.001^*^

^a^ Statistical significance of t-test, analysis of variance, or chi-square, as appropriate.

^*^ Statistically significant at *p* < 0.05.

TBI, traumatic brain injury; M, male; F, female; NSI, Neurobehavioral Symptom Inventory; PCL-5, Post-Traumatic Stress Disorder Checklist for Diagnostic and Statistical Manual of Mental Disorders, Fifth Edition; HIT-6, Headache Impact Test-6.

### MRI data acquisition

Imaging was performed using a Philips Ingenia 3T MRI running software R5.3.1 with a 16-channel matrix head coil. A T1-weighted high-resolution image was acquired using a 3D T1w Turbo Field Echo (TFE) pulse sequence (TFE factor = 256, repetition time (TR) = 6.7 msec, inversion time (TI) = 890 msec, TE = 3.0 msec, shot interval time = 3000 msec, 218 shots, flip angle = 8°, 256 × 256 matrix, phase encoding direction = y). One hundred and seventy slices covering the entire brain were acquired with a voxel resolution of 0.94 × 0.89 × 1 mm. Total scan time was 10:57. Diffusion tensor images were acquired with a pulsed gradient-spin echo sequence with an echo-planar imaging readout in 32 directions (TR = 6853 msec, TE = 103 msec, acquisition matrix = 112 × 110, slice thickness = 3.00 mm, flip angle = 90°, EPI factor = 55). Forty-eight slices covering the entire brain were acquired with an inplane resolution of 2 × 2.04 × 3 mm. Total scan time was 8:46.

### MRI data analysis

#### Structural MRI analysis

MRI structural images were first analyzed using FSL's fMRI analysis tool, FEAT version 6.0.1 (FMRIB's Software Library http://fsl.fmrib.ox.ac.uk/fsl/fslwiki/).^62-64^ The skull was removed from the T1 images using the BET brain extraction tool^65^ with a fractional intensity thresholding of 0.4, specifying the voxel that represented the approximate center of the brain. Next, the T1 was registered to the standard MNI atlas with a 12 degrees of freedom affine transformation. This transformation was refined using FNIRT nonlinear registration with a warp resolution of 10 mm.^66^

Cortical reconstruction and volumetric segmentation were performed with the FreeSurfer image analysis suite (http://surfer.nmr.mgh.harvard.edu), using the built-in command *recon-all* with default settings. The technical details of these procedures are described in prior publications.^45,67–79^ Processing included motion correction, removal of non-brain tissue using a hybrid watershed/surface deformation procedure,^69^ automated Talairach transformation, segmentation of the subcortical white matter and deep gray matter volumetric structures,^45,75^ intensity normalization,^80^ tessellation of the gray matter white matter boundary, automated topology correction,^74,81^ and surface deformation following intensity gradients to optimally place the gray/white and gray/cerebrospinal fluid borders at the location where the greatest shift in intensity defines the transition to the other tissue class.^67,72,73^ Once the cortical models were complete, surface inflation^77^ was performed to match cortical geometry across subjects.^78^ A variety of surface-based data including maps of curvature and sulcal depth were created using both intensity and continuity information from the entire three-dimensional magnetic resonance volume. Cortical thickness was calculated as the closest distance from the gray/white boundary to the gray/CSF boundary at each vertex on the tessellated surface.^73^ The maps were created using spatial intensity gradients across tissue classes and were therefore not simply reliant on absolute signal intensity. Volume, surface area, and thickness for the Desikan-Killiany atlas cortical structures, as well as volume for subcortical structures and white matter segmentations, were extracted for each subject and scaled by total intercranial volume, where appropriate.

#### Voxel-wise DTI analysis of fractional anisotropy

DTI data were analyzed using FSL's FDT toolbox. Voxel-wise statistical analysis of FA was carried out using Tract-Based Spatial Statistics (TBSS),^82^ within FSL.^63^ First, FA images were created by fitting a tensor model to the raw diffusion data using FDT, and then brain-extracted using BET.^65^ All subjects' FA data were then aligned into a common space using the nonlinear registration tool FNIRT,^66,83^ which uses a b-spline representation of the registration warp field.^84^ Next, the mean FA image was created and thinned to create a mean FA skeleton with a threshold FA value of 0.2, representing the centers of all tracts common to the group. Each subject's aligned FA data was then projected onto this skeleton and the resulting data were fed into voxel-wise cross-subject statistics. Statistical analysis was performed using the “randomize” command in FSL. The number of permutations was set to 10,000, and correction for multiple comparisons was achieved using threshold-free cluster enhancement with a family-wise error rate of *p* < 0.05.

#### Region of interest DTI analysis of fractional anisotropy

To evaluate average FA in pre-defined white matter regions of interest, the brain was separated into 48 white matter regions based on the ICBM-label atlas.^85-87^ Average FA was extracted for each region for each participant using the mean FA image created in the FDT process described above. Following FA value extraction, the three participant groups were compared using an analysis of variance (ANOVA) to determine regions of significant difference.

#### Pothole DTI analysis

Because of the heterogeneous nature of white matter damage in TBI, additional analyses were implemented examining DTI “potholes.” Potholes represent white matter with FA values that are abnormally low compared with a healthy control group. Pothole analysis has been shown to be sensitive to white matter changes,^88^ specifically in mTBI, in previous studies.^31,89,90^ To begin, a normative template based on the MNI-registered DTI volumes of the noTBI participants was generated using tools available within FSL. FSL FAST^91^ results were used to restrict the calculations to white matter voxels common among noTBI participants. Volumes representing the mean and standard deviation (SD) of each white matter voxel were then calculated via fslmaths. For each TBI participant, their own FAST white matter map was used to restrict their DTI volume to white matter, and a z-score map was generated by subtracting the control group mean volume and dividing by the control group SD volume. The resulting z-maps were thresholded at z = -3, and FSL cluster was used to label contiguous voxels below this threshold. These resulting clusters are considered potholes. These potholes were then thresholded at ≥10 mm^3^.^90^ The sum of pothole clusters for each participant was then used as the measure of reduced white matter organization in further analyses.

### Statistical analysis

SPSS 28.0 software (IBM Corp., Armonk, NY, USA) was used for statistical analysis. An independent samples t-test or Mann-Whitney test was used to compare group differences depending on data normality. A one-way ANOVA was used when comparing differences among all 3 groups, with *post hoc* Bonferroni analysis for significant difference between two-group pairs. Chi-square analyses were used to compare groups on categorical variables. Effect sizes (d) were computed to demonstrate the magnitude of observed differences. Spearman's correlation coefficients were used to examine associations between white matter and gray matter metrics. The significance level was adjusted by using the Bonferroni correction with *p* < 0.05.

## Results

### Participant demographic and clinical characteristics

Participant characteristics for the three study groups are detailed in Table 1. The proportion of men and women in the study matched expectations, given the sex breakdown in this military population as a whole. There was no significant difference in the distribution of men and women among the three groups [χ^2^(2,90) = 3.289, *p* = 0.193]. Age varied significantly among the three groups [F(2,87) = 9.404, *p* < 0.001, η^2^ = 0.178], with msTBI participants being the youngest overall, followed by the noTBI group, with the mTBI participants being the oldest. Age was controlled in all volumetric analyses. Number of lifetime TBIs ranged from one to 13 and differed between the mTBI and msTBI groups [t(67) = 2.953, *p* = 0.004, d = 0.825]. Sensitivity analyses controlling for repeated injuries were performed, but the results did not meaningfully differ; therefore, this variable is not controlled in further analyses. Time since injury in the mTBI group was significantly greater than in the moderate-severe group [t(67) = 3.413, *p* < 0.001, d = 0.954]. Causes of participants' most recent TBIs varied, including motor vehicle accidents, military training and deployment, falls and accidents, sports-related injuries, and incidences of assault or abuse. There was a significant overall difference between the mTBI and msTBI groups in cause of injury [χ^2^(4,69) = 10.437, *p* = 0.034], with greater observed proportions of mTBIs caused by military activity and sports injuries, and a greater proportion of moderate-severe TBIs caused by accidents/falls. The three groups did not differ significantly in race/ethnicity [χ^2^(12,90) = 9.963, *p* = 0.619]. Average years of education was significantly different among the three groups [F(2,87) = 4.229, *p* = 0.018, η^2^ = 0.089], driven by slightly fewer years of education in the msTBI group. The NSI score differed across the three groups [F(2,87) = 17.443, *p* < 0.001, η^2^ = 0.286], with lowest symptom severity in the noTBI group, followed by the msTBI group and greatest severity of symptoms in the mTBI group. PCL-5 total scores differed among the three groups [F(2,87) = 8.635, *p* < 0.001, η^2^ = 0.174], driven by a *post hoc* significant difference between the noTBI and mTBI groups. Headache Impact Test (HIT-6) total scores also differed among the 3 groups [F(2,87) = 23.422, *p* < 0.001, η^2^ = 0.364], driven by *post hoc* significant differences between the mTBI group and both other groups.

### Voxel-wise DTI analysis of fractional anisotropy

**FIG. 1.**
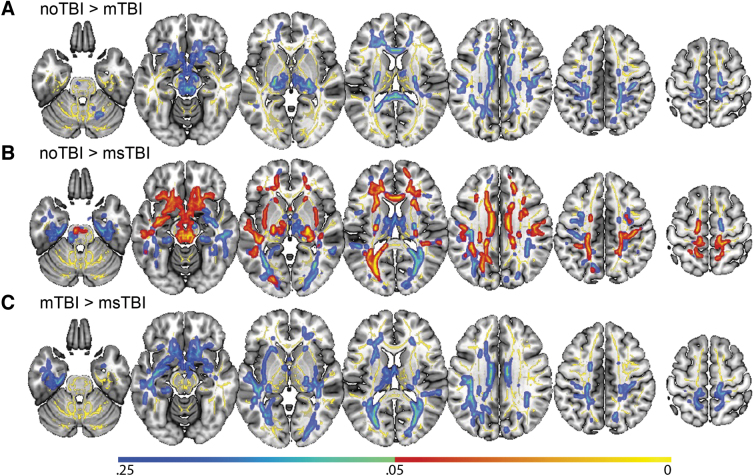
Differences in whole-brain voxel-wise fractional anisotropy (FA) by group. No significant voxel-wise whole brain differences were found between the mild traumatic brain injury (mTBI) group and either of the other two groups. **(A)** Regions with lower FA in the mTBI group compared with the noTBI group; these differences did not reach statistical significance. **(B)** Statistically significant differences in FA between the noTBI group and the moderate or severe TBI (msTBI) group. **(C)** Regions with lower FA in the msTBI group compared with the mTBI group; these differences did not reach statistical significance. Average skeletonized white matter, to which the analysis was restricted, is represented in yellow. Regional differences between groups have been “thickened” for visualization purposes to fill the skeleton into the local white matter tract, and are represented in warm colors (significant at *p* < 0.05) and cool colors (non-significant, 0.25 < *p* < 0.05).

In a voxel-wise analysis of white matter organization, there were no significant differences in FA between the mTBI group and either the noTBI or msTBI groups (Fig. 1A, 1C). There were, however, many areas in which the msTBI group had lower FA than the noTBI group, including voxels in bilateral anterior and superior corona radiata, bilateral external capsule, bilateral anterior limb of internal capsule, left superior longitudinal fasciculus, left posterior corona radiata, bilateral cingulum, left posterior limb and retrolenticular limb of the internal capsule, left posterior thalamic radiation, left sagittal striatum, the full length of the corpus callosum, and bilateral uncinate fasciculus, cerebral peduncle and corticospinal tract (Fig. 1B, warm colors). For display purposes, non-significant differences between groups (0.25 > *p* > 0.05) are presented in cool colors.

### Region of interest DTI analysis of fractional anisotropy

**FIG. 2.**
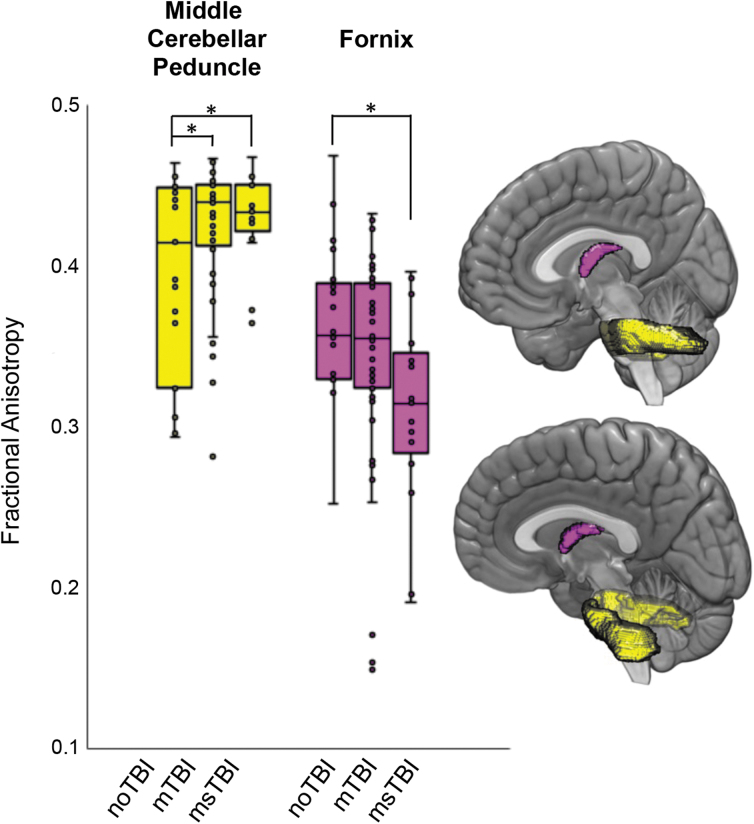
Differences in regional fractional anisotropy (FA) by group. Regions with differential fractional anisotropy among the three groups included the middle cerebellar peduncle (yellow) and the fornix (magenta). Box plots showing the distribution of FA values within each group are presented for the two regions with difference in FA as defined by the ICBM atlas. *post hoc p < 0.05

Separating the brain into 48 white matter regions based on the ICBM-label atlas, two regions were identified where FA values differed significantly among groups. In the middle cerebellar peduncle (Fig. 2, yellow), there was a significant difference among the three groups in regional FA [F(2,87) = 3.562, *p* = 0.033, η^2^ = 0.077], driven by significant *post hoc* differences in average FA between the noTBI group (0.40 ± 0.06) and the two TBI groups (mTBI: 0.42 ± 0.06; msTBI: 0.43 ± 0.03). In the fornix (Fig. 2, magenta), there was a significant difference among the 3 groups in regional FA [F(2,87) = 3.824, *p* = .026, η^2^ = 0.082], driven by *post hoc* significant difference in average FA between the noTBI group (0.37 ± 0.05) and the msTBI group (0.31 ± 0.06).

### Pothole DTI analysis

**FIG. 3.**
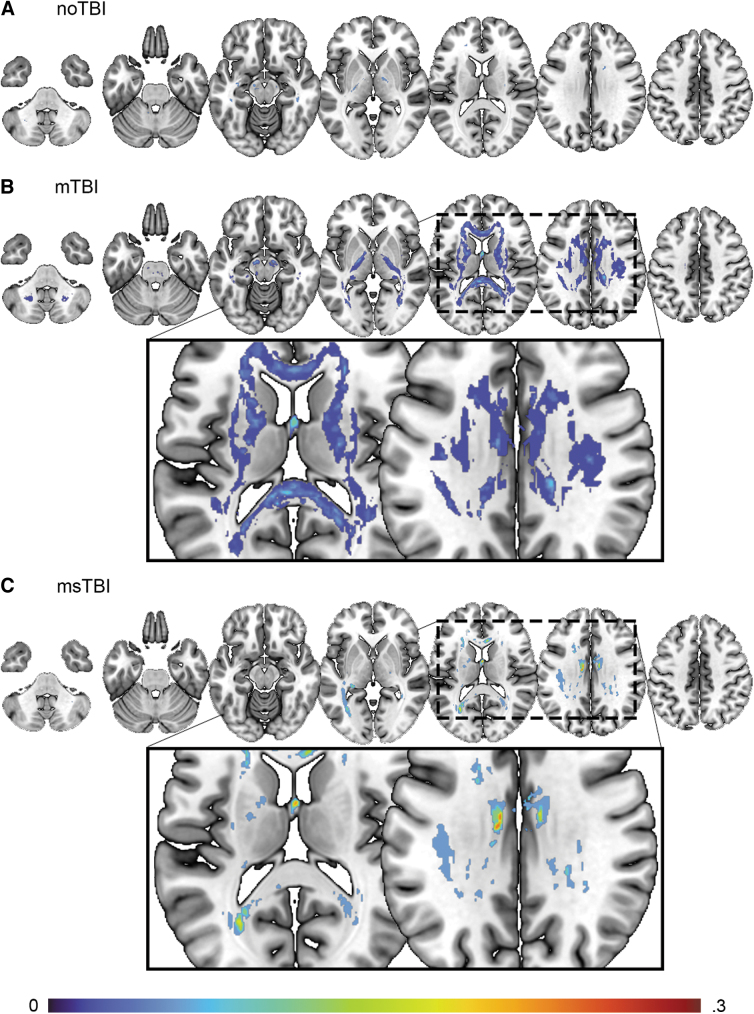
Distribution of potholes for **(A)** noTBI, **(B)** mild traumatic brain injury (mTBI), and **(C)** moderate or severe TBI (msTBI) groups. Colors represent percentage of subgroup population with voxel-wise potholes at Z < 3 below the mean, with a cluster size of at least 10 voxels. Pothole analysis was masked by the white matter tract anatomical regions of interest in the ICBM-label atlas.

Next, clusters with abnormally reduced FA (DTI “potholes”) were identified for each individual, as defined by FA >3 SD below the noTBI population average. Individual participants in the noTBI control group demonstrated a relatively low frequency and small size of DTI potholes, relative to the broader noTBI group, spread in small clusters across the white matter (Fig. 3A). In the mTBI group, potholes were identified in each segment of white matter in the ICBM atlas, with 35% of the mTBI group demonstrating potholes in each of the fornix, corpus callosum, bilateral superior longitudinal fasciculus, left external capsule, right cerebral peduncle, and bilateral internal capsule (Fig. 3B). Potholes in the msTBI group were located in corpus callosum, bilateral cingulum, superior, anterior, and posterior corona radiata, posterior thalamic radiation, superior longitudinal fasciculus, internal capsule, stria terminalis, left sagittal stratum, superior fronto-occipital fasciculus, external capsule, pontine crossing tract, medial lemniscus, and middle cerebellar peduncle (Fig. 3C). These potholes were less widely distributed than the mTBI group, potentially related to the smaller number of msTBI relative to mTBI participants.

**FIG. 4.**
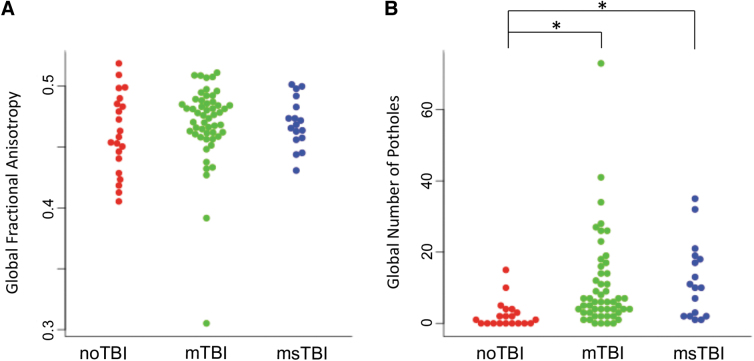
Whole–brain white matter organization. **(A)** There was no difference among groups in average global fractional anisotropy (FA). **(B)** There was a significant difference in number of potholes between the noTBI group (red) and both the mild traumatic brain injury (mTBI) group (green) and moderate or severe TBI (msTBI) group (blue). *p < 0.05

Global number of potholes across the brain differed significantly by group [F(2,87) = 4.642, *p* = 0.012, η^2^ = 0.097]. *Post hoc,* this result was driven by significant differences between the noTBI group (2.45 ± 3.87) and both the mTBI group (10.60 ± 12.93, *p* = 0.019) and the msTBI group (12.00 ± 10.54, *p* = 0.032; Fig. 4B). Average volume of pothole clusters did not differ between groups (*p* = 0.513).

### Cortical segmentation—white matter volume

**FIG. 5.**
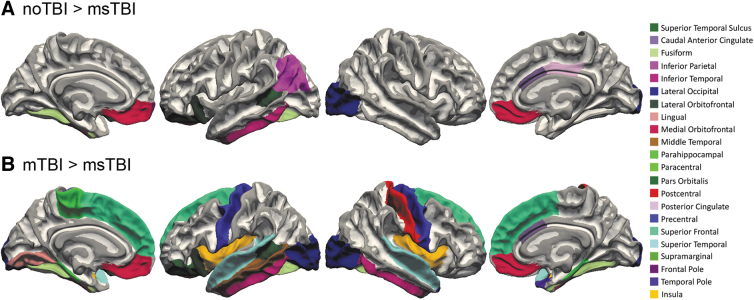
White matter segmentation differences. White matter segmentations with smaller volume in the moderate or severe traumatic brain injury (msTBI) group compared with the **(A)** noTBI group and **(B)** mildTBI (mTBI) groups.

The msTBI group demonstrated significantly less cerebral white matter volume compared with the other two groups [F(2,87) = 8.602, *p* < 0.001, η^2^ = 0.165]. Many individual segmentations of white matter showed decreased volume in the msTBI group compared with the other two groups, including the left superior temporal sulcus, fusiform, inferior temporal, lateral orbitofrontal, right caudal anterior cingulate, lateral occipital, and bilateral medial orbitofrontal white matter. Compared with the noTBI group, msTBI had smaller volume in left inferior parietal, frontal pole, and right posterior cingulate white matter (Fig. 5A). Compared with the mTBI group, the msTBI group showed decreased volume in left lateral occipital, lingual, middle temporal, paracentral, pars orbitalis, and right fusiform, inferior temporal, postcentral, temporal pole, and bilateral parahippocampal, precentral, superior frontal, superior temporal, and insular white matter (Fig. 5B).

### Cortical segmentation—gray matter thickness

**FIG. 6.**
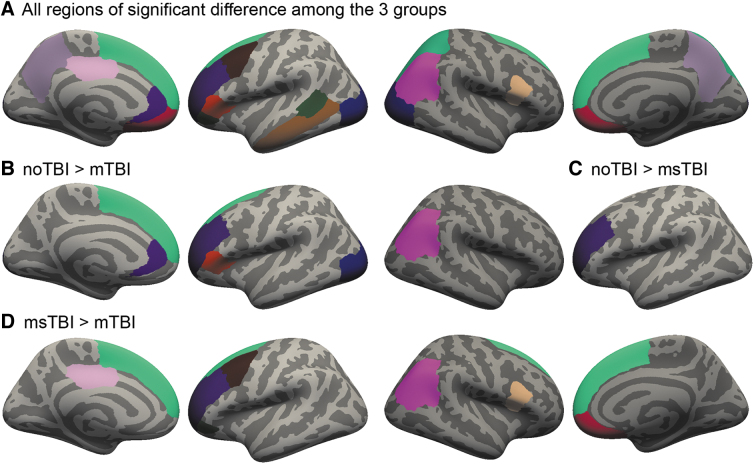
Cortical thickness differences among noTBI, mild traumatic brain injury (mTBI), and moderate or severe TBI (msTBI) groups. FreeSurfer segmentation regions where gray matter thickness differed among noTBI, mTBI, and msTBI groups. **(A)** All regions of significant difference among the three groups. **(B)** Five regions including left lateral occipital gyrus, pars triangularis, rostral anterior cingulate, and superior frontal gyrus were thicker in the noTBI group than in the mTBI group, **(C)** left rostral middle frontal gyrus was thicker in the noTBI group than the msTBI group, and **(D)** five regions including left superior frontal gyrus, rostral middle frontal gyrus, and pars orbitalis, and right medial orbitofrontal gyrus and pars opercularis were thicker in the msTBI group than in the mTBI group.

Gray matter thickness differed significantly among the three groups (η^2^ = 0.069-0.138) in 19 regions, including the left posterior superior temporal sulcus, caudal middle frontal gyrus, middle temporal gyrus, pars orbitalis, pars triangularis, posterior cingulate, rostral anterior cingulate, rostral middle frontal gyrus, right pars opercularis, superior and inferior parietal gyrus, and bilateral lateral occipital gyrus, medial orbitofrontal gyrus, precuneus, and superior frontal gyrus (Fig. 6A). Of these regions, 12 showed *post hoc* differences between at least two groups driving the overall group significance. The noTBI group had thicker gray matter than the mTBI group in left lateral occipital gyrus, pars triangularis, rostral anterior cingulate, superior frontal gyrus, and right inferior parietal gyrus (Fig. 6B). The noTBI group had thicker gray matter than the msTBI group in left rostral middle frontal gyrus (Fig. 6C). The msTBI group had thicker gray matter than the mTBI group in left caudal middle frontal gyrus, pars orbitalis, posterior cingulate, rostral middle frontal gyrus, inferior parietal gyrus, bilateral superior frontal gyrus, right medial orbitofrontal gyrus, and pars opercularis (Fig. 6D).

**FIG. 7.**
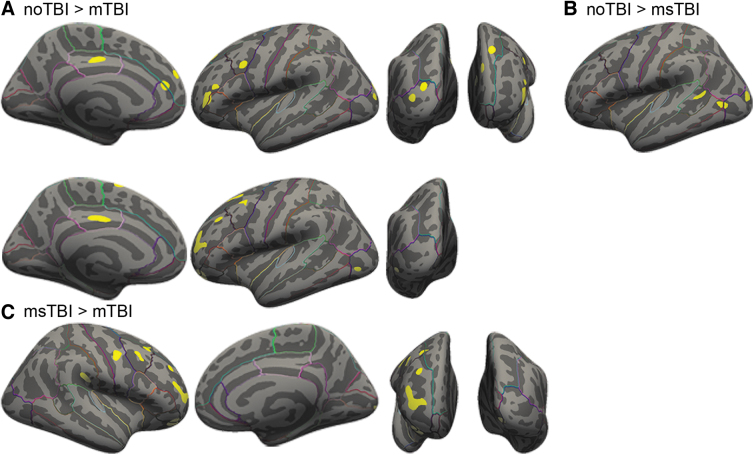
Voxel-wise clusters of gray matter thickness differences. **(A)** Clusters of thicker gray matter in the noTBI group compared with the mild traumatic brain injury (mTBI) group. **(B)** Clusters of thicker gray matter in the noTBI group compared with the msTBI group. **(C)** Clusters of thicker gray matter in the msTBI group compared with the mTBI group.

In a whole–brain voxel-wise comparison of gray matter thickness, there were significant clusters of thickness differences between the noTBI and TBI groups (always noTBI > TBI), and also between the two TBI groups (always msTBI > mTBI). These clusters are depicted in Figure 7 and detailed in Table 2.

**Table 2.** Clusters of Differential Cortical Gray Matter Thickness Between Groups

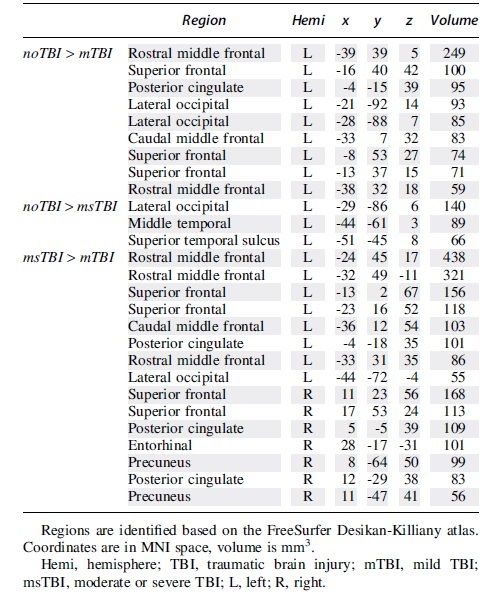

### Sub-cortical segmentation—gray matter volume

Subcortically, volume differed between groups in left thalamus [F(2,87) = 4.249, *p* = 0.017, η^2^ = 0.089] and bilateral accumbens [L: F(2,87) = 6.582, *p* = 0.002, η^2^ = 0.131; R: F(2,87) = 6.126, *p* = 0.003, η^2^ = 0.123]. In the left thalamus, this finding was driven by greater volume in the msTBI group versus the noTBI group. In the left accumbens, the difference was driven by the msTBI group having smaller volume than both the noTBI and mTBI groups, and right accumbens was driven by smaller volume in the msTBI group compared with the mTBI group.

### White and gray matter correlations

Across the full sample, white matter volume was positively correlated with total gray matter volume (*r* = 0.363, *p* < 0.001) and global FA (*r* = 0.301, *p* = 0.004) and negatively correlated with both number of potholes (*r* = -0.227, *p* = 0.009) and global pothole volume (*r* = -0.243, *p* = 0.022). Global FA was negatively correlated with both number of potholes (*r* = -0.484, *p* < 0.001) and global pothole volume (*r* = -0.440, *p* < 0.001).

In the noTBI and mTBI groups, the correlation between cerebral white matter volume and total gray matter volume was maintained (noTBI: *r* = 0.444, *p* = 0.044; mTBI: *r* = 0.420, *p* = 0.002). In each of the three groups, the correlations between global FA and pothole volume (noTBI: *r* = -0.862, *p* < 0.001; mTBI: *r* = -0.439, *p* = 0.002; msTBI: *r* = -0.694, *p* < 0.001) and number of potholes (noTBI: *r* = -0.870, *p* < 0.001; mTBI: *r* = -0.522, *p* = 0.002; msTBI: *r* = -0.713, *p* < 0.001) were maintained. Additionally, in the msTBI group, cerebral white matter volume was negatively correlated with pothole volume (*r* = -0.531, *p* = 0.028) and cluster count (*r* = -0.488, *p* = 0.047) and positively correlated with global average FA (*r* = 0.507, *p* = 0.038). In this group, total gray matter volume was also correlated with pothole cluster count (*r* = -0.568, *p* = 0.017) and global average FA (*r* = 0.525, *p* = 0.031).

### Symptomatology correlation with white and gray matter thickness

The NSI was used as a measure of symptom severity. In the full sample, total NSI score was positively correlated with number of brain-wide potholes (*r* = 0.266, *p* = 0.012) and negatively correlated with global mean gray matter thickness (*r* = -0.234, *p* = 0.026), likely driven by the significant correlation in the mTBI group (*r* = -0.298, *p* = 0.034). NSI total score was not, however, correlated with average global FA (*p* = 0.517). Total score for the PCL-5 was negatively correlated with global mean gray matter thickness, (*r* = -0.254, *p* = 0.019), likely driven by the significant correlation in the mTBI group (*r* = -0.369, *p* = 0.011). PCL-5 total score was also positively correlated with number of brain-wide potholes (*r* = 0.319, *p* = 0.003). The total score for the HIT-6 was negatively correlated with global mean gray matter thickness (*r* = -0.312, *p* = 0.004), driven in part by the significant correlation in the mTBI group (*r* = -0.302, *p* = 0.039). HIT-6 total score was also correlated with global average FA (*r* = 0.228, *p* = 0.036).

## Discussion

This study compared whole-brain DTI and volumetric MRI among 90 Active Duty Service Members with chronic mild TBI (mTBI), chronic moderate-to-severe TBI (msTBI), and TBI-negative controls (noTBI). Results demonstrated that history of TBI was associated with differences in white matter microstructure, white matter volume, and cortical thickness. These white and gray matter abnormalities were also associated with greater severity of self-reported symptoms. However, the frequency, pattern, and neuroanatomical distribution of these MRI findings varied substantially by severity of injury.

DTI has shown promise as an advanced MRI technique to detect potentially-subtle white matter changes related to TBI; however, results of previous studies have been somewhat inconsistent (especially for chronic mTBI). Lacking consensus regarding its utility for diagnosis, patient characterization, and treatment planning, DTI has not yet achieved widespread use in clinical settings. Examination of white matter FA in this study provides an illustration of the varying sensitivity of this imaging modality across different levels of analysis, from aggregated/global white matter FA, to pre-defined white matter ROIs, *post hoc* voxelwise clusters, and quantification of subject-specific FA potholes.

In the most aggregated form (average global FA), DTI results did not differ between mTBI, msTBI, and noTBI groups, and no trends were apparent on plots. Global FA was also unrelated to self-reported neurobehavioral symptoms. However, examination of DTI FA within individual, pre-defined white matter ROIs provided evidence of decreased FA within the fornix of the msTBI group relative to controls, and increased FA within the middle cerebellar peduncle for mTBI and msTBI groups relative to controls. Additionally, voxelwise comparison of DTI white matter FA by group demonstrated widely distributed clusters of reduced FA in the msTBI group relative to controls, but no significant voxelwise findings in the mTBI group. While decreased FA is often interpreted as reflecting axonal injury,^28^ increases in FA may represent axonal swelling or effects of compensatory neuroplasticity.^92^ Collectively, these spatially-defined DTI analyses readily identified altered white matter organization within the msTBI group, but did not provide clear evidence of abnormalities within the mTBI group.

There is reason to believe that white matter organization following TBI may differ between civilian and military populations. This could be due to the mechanism of injury, repeated injury, and the choice of comparison groups. A recent meta-analysis separating studies into civilian, military, and sports-related mTBIs in the acute, subacute, and chronic stages post-injury demonstrated that nine out of 12 studies of civilians with chronic mTBI found reduced FA in white matter tracts throughout the brain.^93^ In a military population, however, reduced FA was only found in six out of 15 studies. Regions of lower FA in this military group included the corpus callosum, superior and inferior longitudinal fasciculus, corona radiata, cingulate, inferior fronto-occipital fasciculus, internal capsule, anterior thalamic radiations, forceps minor and major, corticospinal tract, tapetum, middle cerebellar peduncle, uncinate fasciculus, and cingulum.^94–99^ The current study falls into the camp of the 11 studies that did not find voxelwise differences in FA between mTBI and non-injured groups. In our population, this could be due to our mTBI group having more remote injury than our msTBI group, since our msTBI group did not show significant FA decreases in any specific brain areas relative to our noTBI group. Additionally, because individuals with severe or chronic impairment are likely to separate from active duty, it is possible that the mTBI and msTBI groups in the current sample may be less severely impacted than individuals drawn from other populations.

However, in contrast to the results of our more conventional, spatially-defined DTI analyses, identification of spatially heterogeneous areas of decreased FA (i.e., pothole analysis) provided unique evidence regarding white matter organization in the mTBI group. In the current study, DTI FA potholes were widely distributed through the white matter in both the mTBI and msTBI groups, with elevated prevalence relative to controls. These DTI FA potholes were also related to greater severity of self-reported neurobehavioral symptoms and post-traumatic stress. Importantly, the elevated rates of DTI FA potholes were driven by a subset of the mTBI group, as many mTBI participants had few or no FA potholes. This study joins previous work in military populations showing the utility of pothole measures as potentially-sensitive biomarkers of axonal injury at chronic stages of TBI recovery.^90,100^ However, it remains unclear whether presence, frequency or distribution of FA potholes may simply provide greater sensitivity to TBI relative to traditional DTI analyses, or whether it may represent a distinct biomarker with unique clinical significance relative to mean FA within a given white matter region.

In addition to examining white matter microstructure, analyses also examined white matter volume, with results demonstrating decreased white matter volume in many brain regions for the msTBI group, compared with the other two groups. Previous work, including studies of in vivo neuroimaging and postmortem brain specimens, has shown that white matter degradation persists for many decades post-injury. Postmortem studies of severe TBI patients compared with matched controls found reactive microglia present up to 18 years post-injury. This inflammatory pathology was accompanied by ongoing white matter degradation, including a 25% reduction in corpus callosum thickness.^101^ These results have been replicated in both humans^102,103^ and animal models.^104-106^

Examining cortical thickness across the brain, this study found differences among the three groups, along with greater severity of self-reported neurobehavioral symptoms, post-traumatic stress, and headaches among those with greater cortical thinning. Gray matter differences were most prominent within the mTBI group, in frontal and posterior regions, and in msTBI in the middle frontal gyrus. The results of this study are in line with previous findings, with aggregate evidence suggesting that fronto-temporal areas may be most vulnerable to mTBI;^33^ specifically, this finding of orbitofrontal thinning fits into previous work noting thinning in this region in the mTBI group.^107,108^ Similar to one previous study conducted in a military population,^109^ the current study also provided evidence that mTBI can also be associated with alterations in thickness of the cingulate cortex. Since previous studies have confirmed the stability of these volumetric changes over a 5-year post-injury timeframe,^110^ cortical thickness may be a viable biomarker for assessing long-term effects of injury.

Volumetric analysis also identified areas in which cortical thickness was greater in the msTBI group relative to the mTBI group. However, it is notable that there were no areas with significantly greater cortical thickness in TBI groups relative to noTBI controls. Additionally, out of 48 total ROIs, one region (middle cerebellar peduncle) demonstrated greater FA in the msTBI group relative to noTBI controls. Considering the total number of comparisons, one or more of these counterintuitive findings may represent Type I statistical error. Alternately, these findings may reflect different trajectories of recovery/adaptation for individual moderate-severe injuries, in comparison to the repetitive injuries that were common in our mTBI group. For example, it is plausible that repetitive mTBI could interfere with some aspects of compensatory neuroplasticity that may be preserved in single-incident msTBI.

This study is among the relatively small number of DTI or volumetric MRI studies of TBI that includes data from the full spectrum of injury severity (mild, moderate, and severe TBI). This design provides a number of important advantages for interpretation of data. First, the inclusion of results from chronic msTBI (a group which might be expected to exhibit clear findings on TBI-relevant metrics) provides a point of comparison for mTBI findings, which might otherwise be difficult to interpret in terms of distinguishing statistical versus clinical significance. Additionally, results from this study showed that on some metrics within this population (such as number of DTI FA potholes), chronic-stage mTBI and msTBI may demonstrate similarities to one another, in contrast to uninjured controls. As discussed above, comparisons between these two populations might also provide clues about mechanisms of injury and recovery that can differ based on injury severity.

There are several limitations inherent to studies of TBI, given the heterogenous nature of the injury and the different populations in which TBIs are most common. This study addresses some of these limitations by separating injury into groups of mild and moderate/severe, and limiting participation to those in the chronic stage of injury (> 3 months). Our sample included many more men than women in all groups, though our ratio of men to women mirrors the breakdown found in this military population. Additionally, statistical controls for intracranial volume and age were used where appropriate to manage this potential confound. Results may also have been influenced by the Active Duty military setting from which participants were obtained. For example, the number of total TBIs in our mTBI group was higher than would be expected in a civilian (non-athlete) population, and since permanent/major disability typically results in a medical discharge from active military service, our msTBI group may represent a higher-functioning group than msTBI samples obtained from other settings.

Additionally, mirroring severity prevalence within the TBI population, our msTBI group was smaller than our mTBI group, and had twice as many moderate as severe TBI participants. The moderate and severe groups were combined in this analysis after determining that differences in brain metrics were non-significant between the two groups. Medication, alcohol, and nicotine use were not controlled for in this study, though certain classes of medication were exclusionary for participation. Blast exposure was not controlled for in this study. Since all participants are from an Active Duty military population, it stands to reason that many participants will have had blast exposure in addition to their TBIs. Blast exposure should be examined more systematically in future studies in this population. The addition of an orthopedic injury control group in future studies may also aid in our understanding of TBI effects on white matter microstructure and gray matter integrity. Finally, the problem of crossing fibers is fundamental in DTI due to FA being artificially suppressed within multi-directional fiber tracts. In future work we hope to acquire scans with increased resolution and directions to resolve crossing fibers and achieve increased sensitivity to potential abnormalities in these white matter tracts.

## Conclusions

This study of whole–brain DTI and volumetric MRI in Active Duty Service Members with chronic TBI demonstrated that a history of TBI was associated with differences in white matter microstructure, white matter volume, and cortical thickness in both mild and moderate-to-severe TBI groups relative to uninjured controls. These white and gray matter abnormalities were also associated with greater severity of self-reported symptoms. Further exploration showed that the presence, pattern and neuroanatomical distribution of MRI findings varied substantially by severity of injury. This study expands upon a growing body of literature using advanced imaging techniques to identify potential effects of brain injury in military service members.

## Transparency, Rigor, and Reproducibility Summary

This study was not formally registered because it is not a clinical trial. The analysis plan was not formally pre-registered, but the team member with primary responsibility for the analysis (lead author) certifies that the analysis plan was pre-specified. Sample size was 21 healthy controls, 52 mTBI, and 17 msTBI subjects, and the observed effect sizes were moderate. Ninety-eight participants were enrolled in MRI portion of the study and had images collected. Ninety participants had images that passed systematic quality assessments and were analyzed. Imaging quality control decisions and analyses were performed by investigators who were aware of relevant characteristics of the participants. Imaging data were acquired between February 2018 and May 2022 between 12-2 p.m. Imaging data were collecting using a Philips Ingenia 3T MRI running software R5.3.1 with a 16-channel matrix head coil. All imaging was collected using the same scanner. The time required for image acquisition was 48 min. Complete imaging parameters are presented in the methods. All equipment and software used to perform acquisition and analysis are widely available from Philips and FSL. The key inclusion criteria (TBI diagnostic criteria) are established standards in the field. Correction for multiple comparisons was performed within FSL as described in the methods. No replication or external validation studies have been performed or are planned/ongoing at this time to our knowledge. De-identified data from this study are not available in a public archive. De-identified data from this study will be made available (as allowable according to institutional IRB standards) by emailing the corresponding author. Analytic code used to conduct the analyses presented in this study are not available in a public repository. They may be available by emailing the corresponding author. The authors agree to provide the full content of the manuscript on request by contacting the corresponding author.

## References

[1] TBICoE. Worldwide Numbers for TBI. 2023. Available from: https://www.health.mil/Military-Health-Topics/Centers-of-Excellence/Traumatic-Brain-Injury-Center-of-Excellence/DOD-TBI-Worldwide-Numbers [Last accessed October 27, 2023].

[2] Hulkower MB, Poliak DB, Rosenbaum SB, et al. A decade of DTI in traumatic brain injury: 10 years and 100 articles later. Am J Neuroradiol 2013;34(11):2064–2074; doi: 10.3174/ajnr.A3395 23306011 PMC7964847

[3] Palacios EM, Yuh EL, Donald CL Mac, et al. Diffusion tensor imaging reveals elevated diffusivity of white matter microstructure that is independently associated with long-term outcome after mild traumatic brain injury: a TRACK-TBI study. J Neurotrauma 2022;39:1318–1328; doi: 10.1089/neu.2021.0408 35579949 PMC9529303

[4] Kim E, Yoo RE, Seong MY, et al. A systematic review and data synthesis of longitudinal changes in white matter integrity after mild traumatic brain injury assessed by diffusion tensor imaging in adults. Eur J Radiol 2022;147:110117; doi: 10.1016/J.EJRAD.2021.110117 34973540

[5] Arfanakis K, Haughton VM, Carew JD, et al. Diffusion tensor MR imaging in diffuse axonal injury. AJNR Am J Neuroradiol 2002;23(5):794–802. 12006280 PMC7974716

[6] Bigler ED. Neuroimaging in Mild TBI. In: The Evaluation and Treatment of Mild Traumatic Brain Injury. (Varney NR, Roberts RJ. eds) Lawrence Erlbaum Associates: Mahwah, NJ; 1999; pp. 63–80.

[7] Basser PJ, Pierpaoli C. A simplified method to measure the diffusion tensor from seven MR images. Magn Reson Med 1998;39(6):928–934; doi: 10.1002/mrm.1910390610 9621916

[8] Marmarou A, Signoretti S, Fatouros PP, et al. Predominance of cellular edema in traumatic brain swelling in patients with severe head injuries. J Neurosurg 2006;104(5):720–730; doi: 10.3171/JNS.2006.104.5.720 16703876

[9] Newcombe VFJ, Williams GB, Nortje J, et al. Analysis of acute traumatic axonal injury using diffusion tensor imaging. Br J Neurosurg 2007;21(4):340–348; doi: 10.1080/02688690701400882 17676452

[10] Kennedy MRT, Wozniak JR, Muetzel RL, et al. White matter and neurocognitive changes in adults with chronic traumatic brain injury. J Int Neuropsychol Soc 2009;15(1):130–136; doi: 10.1017/S1355617708090024 19128536 PMC2895769

[11] Kraus MF, Susmaras T, Caughlin BP, et al. White matter integrity and cognition in chronic traumatic brain injury: a diffusion tensor imaging study. Brain 2007;130:2508–2519; doi: 10.1093/brain/awm216 17872928

[12] Nakayama N, Okumura A, Shinoda J, et al. Evidence for white matter disruption in traumatic brain injury without macroscopic lesions. J Neurol Neurosurg Psychiatry 2006;77(7):850–855; doi: 10.1136/jnnp.2005.077875 16574734 PMC2117497

[13] Palacios EM, Sala-Llonch R, Junque C, et al. White matter integrity related to functional working memory networks in traumatic brain injury. Neurology 2012;78(12):852–860; doi: 10.1212/WNL.0B013E31824C465A 22345222

[14] Xu J, Rasmussen IA, Lagopoulos J, et al. Diffuse axonal injury in severe traumatic brain injury visualized using high-resolution diffusion tensor imaging. J Neurotrauma 2007;24(5):753–765; doi: 10.1089/NEU.2006.0208 17518531

[15] Gentry LA, Godersky JC, Thompson B. MR imaging of head trauma: review of the distribution and radiopathologic features of traumatic lesions. AJR Am J Roentgenol 1988;150:663–672; doi: 10.2214/ajr.150.3.663 3257624

[16] Reinarz SJ, Coffman CE, Smoker WAK, et al. MR imaging of the corpus callosum: Normal and pathologic findings and correlation with CT. Am J Roentgenol 1987;151:791–798; doi: 10.2214/ajr.151.4.791 3262282

[17] Tate DF, Bigler ED. Fornix and hippocampal atrophy in traumatic brain injury. Learn Mem 2000; 7:442–446. doi: 10.1101/lm.33000 11112803

[18] Blumbergs PC, Scott G, Vis JM, et al. Topography of axonal injury as defined by amyloid precursor protein and the sector scoring method in mild and severe closed head injury. J Neurotrauma 1995;12(4):565–572; doi: 10.1089/NEU.1995.12.565 8683607

[19] Kinnunen KM, Greenwood R, Hilary Powell J, et al. White matter damage and cognitive impairment after traumatic brain injury. Brain 2011;134:449–463; doi: 10.1093/brain/awq347 21193486 PMC3030764

[20] Mac Donald CL, Johnson AM, Cooper D, et al. Detection of blast-related traumatic brain injury in U.S. military personnel. N Engl J Med 2011;364(22):2091–2100; doi: 10.1056/NEJMOA1008069 21631321 PMC3146351

[21] Warden DL, French LM, Shupenko L, et al. Case report of a soldier with primary blast brain injury. Neuroimage 2009;47 Suppl 2(SUPPL. 2); doi: 10.1016/J.NEUROIMAGE.2009.01.060 19457364

[22] Wallace EJ, Mathias JL, Ward L. Diffusion tensor imaging changes following mild, moderate and severe adult traumatic brain injury: a meta-analysis. Brain Imaging Behav 2018;12(6):1607–1621; doi: 10.1007/s11682-018-9823-2 29383621

[23] Lo C, Shifteh K, Gold T, et al. Diffusion tensor imaging abnormalities in patients with mild traumatic brain injury and neurocognitive impairment. J Comput Assist Tomogr 2009;33(2):293–297; doi: 10.1097/RCT.0B013E31817579D1 19346863

[24] Fakhran S, Yaeger K, Alhilali L. Symptomatic White matter changes in mild traumatic brain injury resemble pathologic features of early alzheimer dementia. Radiology 2013;269:249–257; doi: 10.1148/radiol.13122343 23781117

[25] Geary EK, Kraus MF, Pliskin NH, et al. Verbal learning differences in chronic mild traumatic brain injury. J Int Neuropsychol Soc 2010;1–11; doi: 10.1017/S135561771000010X 20188015

[26] Lipton ML, Gellella E, Lo C, et al. Multifocal white matter ultrastructural abnormalities in mild traumatic brain injury with cognitive disability: a voxel-wise analysis of diffusion tensor imaging. J Neurotrauma 2008;25:1335–1342; doi: 10.1089/neu.2008.0547 19061376

[27] Goetz P, Blamire A, Rajagopalan B, et al. Increase in apparent diffusion coefficient in normal appearing white matter following human traumatic brain injury correlates with injury severity. J Neurotrauma 2004;21(6):645–654; doi: 10.1089/0897715041269731 15253793

[28] Yin B, Li DD, Huang H, et al. Longitudinal changes in diffusion tensor imaging following mild traumatic brain injury and correlation with outcome. Front Neural Circuits 2019;13(May):1–11; doi: 10.3389/fncir.2019.00028 31133818 PMC6514143

[29] Rutgers DR, Toulgoat F, Cazejust J, et al. White matter abnormalities in mild traumatic brain injury: a diffusion tensor imaging study. AJNR Am J Neuroradiol 2008;29:514–519; doi: 10.3174/ajnr.A0856 18039754 PMC8118864

[30] Aoki Y, Inokuchi R, Gunshin M, et al. Diffusion tensor imaging studies of mild traumatic brain injury: a meta-analysis. J Neurol Neurosurg Psychiatry 2012;83:870–876; doi: 10.1136/jnnp-2012-302742 22797288 PMC3415311

[31] Jorge RE, Acion L, White T, et al. White matter abnormalities in veterans with mild traumatic brain injury. Am J Psychiatry 2012;169(12):1284–1291; doi: 10.1176/appi.ajp.2012.12050600 23212059 PMC4030599

[32] Lewén A, Fredriksson A, Li GL, et al. Behavioural and morphological outcome of mild cortical contusion trauma of the rat brain: Influence of NMDA-Receptor blockade. Acta Neurochir (Wien) 1999;141(2):193–202; doi: 10.1007/s007010050286 10189503

[33] Bigler ED. Volumetric MRI Findings in mild traumatic brain injury (mTBI) and neuropsychological outcome. Neuropsychol Rev 2023 Mar;33(1):5–41; doi: 10.1007/s11065-020-09474-0 33656702

[34] Tate DF, Wade BSC, Velez CS, et al. Volumetric and shape analyses of subcortical structures in United States service members with mild traumatic brain injury. J Neurol 2016;263(10):2065–2079; doi: 10.1007/s00415-016-8236-7 27435967 PMC5564450

[35] Govindarajan KA, Narayana PA, Hasan KM, et al. Cortical rhickness in mild traumatic brain injury. J Neurotrauma 2016;33(20):1809–1817; doi: 10.1089/neu.2015.4253 26959810 PMC5079411

[36] Urban KJ, Riggs L, Wells GD, et al. Cortical thickness changes and their relationship to dual-task performance following mild traumatic brain injury in youth. J Neurotrauma 2017;34(4):816–823; doi: 10.1089/neu.2016.4502 27629883

[37] Epstein DJ, Legarreta M, Bueler E, et al. Orbitofrontal cortical thinning and aggression in mild traumatic brain injury patients. Brain Behav 2016;6(12):1–10; doi: 10.1002/brb3.581 PMC5167002 28032004

[38] Fogleman ND, Naaz F, Knight LK, et al. Reduced lateral prefrontal cortical volume is associated with performance on the modified Iowa Gambling Task: a surface based morphometric analysis of previously deployed veterans. Psychiatry Res Neuroimaging 2017;267(June):1–8; doi: 10.1016/j.pscychresns.2017.06.014 28672256

[39] Spitz G, Bigler ED, Abildskov T, et al. Regional cortical volume and cognitive functioning following traumatic brain injury. Brain Cogn 2013;83(1):34–44; doi: 10.1016/j.bandc.2013.06.007 23872098

[40] Tate DF, Brain V, York GE, et al. Preliminary findings of cortical thickness abnormalities in blast injured service members and their relationship to clinical findings. Brain Imaging Behav 2016;8(1):102–109; doi: 10.1007/s11682-013-9257-9 PMC4714342 24100952

[41] Hayes JP, Logue MW, Sadeh N, et al. Mild traumatic brain injury is associated with reduced cortical thickness in those at risk for Alzheimer's disease. Brain 2017;140(3):813–825; doi: 10.1093/brain/aww344 28077398 PMC6075586

[42] Lindemer ER, Salat DH, Leritz EC, et al. Reduced cortical thickness with increased lifetime burden of PTSD in OEF/OIF veterans and the impact of comorbid TBI. NeuroImage Clin 2013;2(1):601–611; doi: 10.1016/j.nicl.2013.04.009 24179811 PMC3777819

[43] Michel BF, Sambuchi N, Vogt BA. Impact of mild traumatic brain injury on cingulate functions. Handb Clin Neurol 2019:166:151–162; doi: 10.1016/B978-0-444-64196-0.00010-8 31731910

[44] Newsome MR, Wilde EA, Bigler ED, et al. Functional brain connectivity and cortical thickness in relation to chronic pain in post-911 veterans and service members with mTBI. Brain Inj 2018;32(10):1236–1244; doi: 10.1080/02699052.2018.1494853 30047797

[45] Fischl B, Salat D ∼H., Busa E, et al. Whole brain segmentation: automated labeling of neuroanatomical structures in the human brain. Neuron 2002;33:341–355; doi: 10.1016/s0896-6273(02)00569-x. 11832223

[46] Salat DH, Robinson ME, Miller DR, et al. Neuroimaging of deployment-associated traumatic brain injury (TBI) with a focus on mild TBI (mTBI) since 2009. Brain Inj 2017;31(9):1204–1219; doi: 10.1080/02699052.2017.1327672 28981347 PMC9206728

[47] Savjani RR, Taylor BA, Acion L, et al. Accelerated changes in cortical thickness measurements with age in military Service Members with traumatic brain injury. J Neurotrauma 2017;34(22):3107–3116; doi: 10.1089/neu.2017.5022 28657432

[48] Shao M, Cao J, Bai L, et al. Preliminary evidence of sex differences in cortical thickness following acute mild traumatic brain injury. Front Neurol 2018;9(OCT):1–7; doi: 10.3389/fneur.2018.00878 30386291 PMC6199374

[49] Bigler ED, Zielinski BA, Goodrich-Hunsaker N, et al. The relation of focal lesions to cortical thickness in pediatric traumatic brain injury. J Child Neurol 2016;31(11):1302–1311; doi: 10.1177/0883073816654143 27342577 PMC5525324

[50] España LY, Lee RM, Ling JM, et al. Serial assessment of gray matter abnormalities after sport-related concussion. J Neurotrauma 2017;34(22):3143–3152; doi: 10.1089/neu.2017.5002 28665173

[51] Wojtowicz M, Gardner AJ, Stanwell P, et al. Cortical thickness and subcortical brain volumes in professional rugby league players. NeuroImage Clin 2018:377–381; doi: 10.1016/j.nicl.2018.01.005 PMC5814377 29487794

[52] Morganti-Kossmann MC, Rancan M, Stahel PF, et al. Inflammatory response in acute traumatic brain injury: a double-edged sword. Curr Opin Crit Care 2002;8(2):101–105; doi: 10.1097/00075198-200204000-00002 12386508

[53] Wang J, Doré S. Inflammation after intracerebral hemorrhage. J Cereb Blood Flow Metab 2007;27(5):894–908; doi: 10.1038/sj.jcbfm.9600403 17033693

[54] Victoroff J, Bigler ED. Concussion and traumatic encephalopathy: causes, diagnosis, and management. Brain Inj 2020;34(7):574–582; doi: 10.1017/9781139696432

[55] Bogner J, Corrigan JD. Reliability and predictive validity of the Ohio State University TBI identification method with prisoners. J Head Trauma Rehabil 2009;24:279–291; doi: 10.1097/HTR.0b013e3181a66356 19625867

[56] Corrigan JD, Bogner J. Initial reliability and validity of the Ohio State University TBI Identification Method. J Head Trauma Rehabil 2007;22:318–329; doi: 10.1097/01.HTR.0000300227.67748.77 18025964

[57] Cicerone KD, Kalmar K. Persistent postconcussion syndrome: the structure of subjective complaints after mild traumatic brain injury. J Head Trauma Rehabil 1995;10:1–17.

[58] Weathers FW, Litz BT, Herman DS, et al. The PTSD Checklist (PCL): Reliability, validity, and diagnostic utility. Annu Meet Int Soc Trauma Stress Stud 1993;1–3.

[59] Rendas-Baum R, Yang M, Varon SF, et al. Validation of the Headache Impact Test (HIT-6) in patients with chronic migraine. Heal Qual Life Outcomes 2014;12:117; doi: 10.1186/s12955-014-0117-0 PMC4243819 25080874

[60] Yang M, Rendas-Baum R, Varon SF, et al. Validation of the Headache Impact Test (HIT-6) across episodic and chronic migraine. Cephalalgia 2011;31:357–367; doi: 10.1177/0333102410379890 20819842 PMC3057423

[61] Kosinski M, Bayliss MS, Bjorner JB, et al. A six-item short-form survey for measuring headache impact: the HIT-6. Qual Life Res 2003;12(8):963–974; doi: 10.1023/A:1026119331193 14651415

[62] Woolrich MW, Ripley BD, Brady M, et al. Temporal autocorrelation in univariate linear modeling of FMRI data. Neuroimage 2001;14(6):1370–1386; doi: 10.1006/nimg.2001.0931 11707093

[63] Smith SM, Jenkinson M, Woolrich MW, et al. Advances in functional and structural MR image analysis and implementation as FSL. Neuroimage 2004;23:S208–S219. 15501092 10.1016/j.neuroimage.2004.07.051

[64] Jenkinson M, Beckmann CF, Behrens TEJ, et al. FSL. Neuroimage 2012;62(2):782–790; doi: 10.1016/J.NEUROIMAGE.2011.09.015 21979382

[65] Smith SM. Fast robust automated brain extraction. Hum Brain Mapp 2002;17(3):143–155; doi: 10.1002/hbm.10062 12391568 PMC6871816

[66] Andersson JLR, Jenkinson M, Smith S. Non-linear registration aka spatial normalisation FMRIB Technial Report TR07JA2. 2007. Available from: https://www.fmrib.ox.ac.uk/datasets/techrep/tr07ja2/tr07ja2.pdf [Last accessed October 27, 2023].

[67] Dale AM, Fischl B, Sereno MI. Cortical surface-based analysis. I. Segmentation and surface reconstruction. Neuroimage 1999;9:179–194; doi: 10.1006/nimg.1998.0395 9931268

[68] Jovicich J, Czanner S, Greve D, et al. Reliability in multi-site structural MRI studies: Effects of gradient non-linearity correction on phantom and human data. Neuroimage 2006;30(2):436–443; doi: DOI: 10.1016/j.neuroimage.2005.09.046 16300968

[69] Segonne F, Dale AM, Busa E, et al. A hybrid approach to the skull stripping problem in MRI. Neuroimage 2004;22(3):1060–1075; doi: DOI: 10.1016/j.neuroimage.2004.03.032 15219578

[70] Reuter M, Rosas HD, Fischl B. Highly accurate inverse consistent registration: A robust approach. Neuroimage 2010;53(4):1181–1196; doi: 10.1016/j.neuroimage.2010.07.020 20637289 PMC2946852

[71] Reuter M, Schmansky NJ, Rosas HD, et al. Within-subject template estimation for unbiased longitudinal image analysis. Neuroimage 2012;61(4):1402–1418; doi: 10.1016/j.neuroimage.2012.02.084 22430496 PMC3389460

[72] Dale AM, Sereno MI. Improved localizadon of cortical activity by combining EEG and MEG with MRI cortical surface reconstruction: a linear approach. J Cogn Neurosci 1993;5(2); doi: 10.1162/JOCN.1993.5.2.162 23972151

[73] Fischl B, Dale AM. Measuring the thickness of the human cerebral cortex from magnetic resonance images. Proc Natl Acad Sci U S A 2000;97:11050–11055; doi: 10.1073/pnas.200033797200033797 10984517 PMC27146

[74] Fischl B, Liu A, Dale AM. Automated manifold surgery: constructing geometrically accurate and topologically correct models of the human cerebral cortex. IEEE Med Imaging 2001;20(1):70–80; doi: 10.1109/42.906426 11293693

[75] Fischl B, Salat DH, van der Kouwe AJW, et al. Sequence-independent segmentation of magnetic resonance images. Neuroimage 2004:23 Suppl 1:S69–S84; doi: 10.1016/j.neuroimage.2004.07.016 15501102

[76] Fischl B, van der Kouwe A, Destrieux C, et al. Automatically parcellating the human cerebral cortex. Cereb Cortex 2004;14(1):11–22; doi: 10.1093/cercor/bhg087 14654453

[77] Fischl B, Sereno MI, Tootell RBH, et al. High-resolution intersubject averaging and a coordinate system for the cortical surface. Hum Brain Mapp 1999;8(4):272–284; doi: 10.1002/(SICI)1097-0193(1999)8:4<272::AID-HBM10>3.0.CO;2-4 10619420 PMC6873338

[78] Fischl B, Sereno MI, Dale A. Cortical surface-based analysis: II: inflation, flattening, and a surface-based coordinate system. Neuroimage 1999;9(2):195–207; doi: 10.1006/nimg.1998.0396 9931269

[79] Han X, Jovicich J, Salat D, et al. Reliability of MRI-derived measurements of human cerebral cortical thickness: the effects of field strength, scanner upgrade and manufacturer. Neuroimage 2006;32(1):180–194; doi: 10.1016/j.neuroimage.2006.02.051 16651008

[80] Sled JG, Zijdenbos AP, Evans AC.. IEEE Trans Med Imaging 1998;17:87–97. 9617910 10.1109/42.668698

[81] Segonne F, Pacheco J, Fischl B. Geometrically accurate topology-correction of cortical surfaces using nonseparating loops. IEEE Trans Med Imaging 2007;26:518–529; doi: 10.1109/42.668698 17427739

[82] Smith SM, Jenkinson M, Johansen-Berg H, et al. Tract-based spatial statistics: voxelwise analysis of multi-subject diffusion data. Neuroimage 2006;31(4):1487–505; doi: 10.1016/j.neuroimage.2006.02.024 16624579

[83] Andersson J, Jenkinson M, Smith S. Non-Linear Optimisation. 2007. Available from: https://www.fmrib.ox.ac.uk/datasets/techrep/tr07ja1/tr07ja1.pdf [Last accessed October 27, 2023].

[84] Rueckert D, Sonoda LI, Hayes C, et al. Nonrigid registration using free-form deformations: application to breast MR images. IEEE Trans Med Imaging 1999;18(8):712–21; doi: 10.1109/42.796284 10534053

[85] Mori S, Wakana S, Nagae-Poetscher LM, Van Zijl, PCM. (eds.) MRI atlas of human white matter. Elsevier: St Louis, MO; 2005.

[86] Wakana S, Caprihan A, Panzenboeck MM, et al. Reproducibility of quantitative tractography methods applied to cerebral white matter. Neuroimage 2007;36(3):630–644; doi: 10.1016/j.neuroimage.2007.02.049 17481925 PMC2350213

[87] Hua K, Zhang J, Wakana S, et al. Tract probability maps in stereotaxic spaces: analyses of white matter anatomy and tract-specific quantification. Neuroimage 2008;32(7):736–740; doi: 10.1016/j.neuroimage.2007.07.053 PMC2724595 17931890

[88] de Mol CL, Neuteboom RF, Jansen PR, et al. White matter microstructural differences in children and genetic risk for multiple sclerosis: a population-based study. Mult Scler J 2022;28(5):730–741; doi: 10.1177/13524585211034826 PMC8978478 34379023

[89] Taber KH, Hurley RA, Haswell CC, et al.. 2015;30(1):1–20; doi: 10.1097/HTR.0000000000000030

[90] Jurick SM, Hoffman SN, Sorg S, et al. Pilot investigation of a novel white matter imaging technique in Veterans with and without history of mild traumatic brain injury. Brain Inj 2018;32(10):1256–1265; doi: 10.1080/02699052.2018.1493225 30169992

[91] Zhang Y, Brady M, Smith S. Segmentation of brain MR images through a hidden Markov random field model and the expectation-maximization algorithm. IEEE Trans Med Imaging 2001;20(1):45–57; doi: 10.1109/42.906424 11293691

[92] Kimura-Ohba S, Yang Y, Thompson J, et al. Transient increase of fractional anisotropy in reversible vasogenic edema. J Cereb Blood Flow Metab 2016;36(10):1731–1743; doi: 10.1177/0271678X16630556 26865662 PMC5076788

[93] Asken BM, DeKosky ST, Clugston JR, et al. Diffusion tensor imaging (DTI) findings in adult civilian, military, and sport-related mild traumatic brain injury (mTBI): a systematic critical review. Brain Imaging Behav 2018;12(2):585–612; doi: 10.1007/s11682-017-9708-9 28337734

[94] Matthews SC, Strigo IA, Simmons AN, et al. A multimodal imaging study in U.S. veterans of Operations Iraqi and Enduring Freedom with and without major depression after blast-related concussion. Neuroimage 2011;54(SUPPL. 1):S69–S75; doi: 10.1016/j.neuroimage.2010.04.269 20451622

[95] Matthews SC, Spadoni AD, Lohr JB, et al. Diffusion tensor imaging evidence of white matter disruption associated with loss versus alteration of consciousness in warfighters exposed to combat in Operations Enduring and Iraqi Freedom. Psychiatry Res 2012;204(2–3):149–154; doi: 10.1016/j.pscychresns.2012.04.018 23149025 PMC3866101

[96] Davenport ND, Lim KO, Armstrong MT, et al. Diffuse and spatially variable white matter disruptions are associated with blast-related mild traumatic brain injury. Neuroimage 2012;59(3):2017–2024; doi: 10.1016/j.neuroimage.2011.10.050 22040736

[97] Morey RA, Haswell CC, Selgrade ES, et al. Effects of chronic mild traumatic brain injury on white matter integrity in Iraq and Afghanistan war veterans. Hum Brain Mapp 2013;34(11):2986–2999; doi: 10.1002/hbm.22117 22706988 PMC3740035

[98] Mac Donald C, Johnson A, Cooper D, et al. Cerebellar white matter abnormalities following primary blast injury in US military personnel. PLoS One 2013;8(2); doi: 10.1371/journal.pone.0055823 PMC3567000 23409052

[99] Petrie EC, Cross DJ, Yarnykh VL, et al. Neuroimaging, behavioral, and psychological sequelae of repetitive combined blast/impact mild traumatic brain injury in Iraq and Afghanistan war veterans. J Neurotrauma 2014;31(5):425–436; doi: 10.1089/neu.2013.2952 24102309 PMC3934596

[100] Jorge RE, Acion L, White T, et al. White matter abnormalities in veterans with mild traumatic brain injury. Am J Psychiatry 2012 Dec;169(12):1284–1291; doi: 10.1176/appi.ajp.2012.12050600 PMC4030599 23212059

[101] Johnson VE, Stewart JE, Begbie FD, et al. Inflammation and white matter degeneration persist for years after a single traumatic brain injury. Brain 2013;136(1):28–42; doi: 10.1093/brain/aws322 23365092 PMC3562078

[102] Chen XH, Johnson VE, Uryu K, et al. A lack of amyloid β plaques despite persistent accumulation of amyloid β in axons of long-term survivors of traumatic brain injury. Brain Pathol 2009;19(2):214–223; doi: 10.1111/j.1750-3639.2008.00176.x 18492093 PMC3014260

[103] Tomaiuolo F, Bivona U, Lerch JP, et al. Memory and anatomical change in severe non missile traumatic brain injury: ∼1 vs. ∼8years follow-up. Brain Res Bull 2012;87(4–5):373–382; doi: 10.1016/j.brainresbull.2012.01.008 22289841

[104] Pierce JES, Smith DH, Trojanowski JQ, et al. Enduring cognitive, neurobehavioral and histopathological changes persist for up to one year following severe experimental brain injury in rats. Neuroscience 1998;87(2):359–369; doi: 10.1016/S0306-4522(98)00142-0 9740398

[105] Chen XH, Siman R, Iwata A, et al. Long-term accumulation of amyloid-β, β-secretase, presenilin-1, and caspase-3 in damaged axons following brain trauma. Am J Pathol 2004;165(2):357–371; doi: 10.1016/S0002-9440(10)63303-2 15277212 PMC1618579

[106] Byrnes KR, Loane DJ, Stoica BA, et al. Delayed mGluR5 activation limits neuroinflammation and neurodegeneration after traumatic brain injury. J Neuroinflammation 2012;9(1):43; doi: 10.1186/1742-2094-9-43 22373400 PMC3308916

[107] Patel JB, Wilson SH, Oakes TR, et al. Structural and volumetric brain MRI findings in mild traumatic brain injury. Am J Neuroradiol 2020;41(1):92–99; doi: 10.3174/ajnr.A6346 31896572 PMC6975320

[108] Sussman D, da Costa L, Chakravarty MM, et al. Concussion induces focal and widespread neuromorphological changes. Neurosci Lett 2017;650:52–59; doi: 10.1016/j.neulet.2017.04.026 28428014

[109] Eierud C, Nathan DE, Bonavia GH, et al. Cortical thinning in military blast compared with non-blast persistent mild traumatic brain injuries. NeuroImage Clin 2019;22:101793; doi: 10.1016/j.nicl.2019.101793 30939340 PMC6446073

[110] Mac Donald CL, Barber J, Andre J, et al. Longitudinal neuroimaging following combat concussion: sub-acute, 1 year and 5 years post-injury. Brain Commun 2019;1(1):1–13; doi: 10.1093/braincomms/fcz031 PMC6935683 31915753

